# Identification of Developmentally Regulated PCP-Responsive Non-Coding RNA, *prt6*, in the Rat Thalamus

**DOI:** 10.1371/journal.pone.0097955

**Published:** 2014-06-02

**Authors:** Hironao Takebayashi, Naoki Yamamoto, Asami Umino, Toru Nishikawa

**Affiliations:** Department of Psychiatry and Behavioral Sciences, Graduate School of Medical and Dental Sciences, Tokyo Medical and Dental University, Bunkyo-ku, Tokyo, Japan; Hokkaido University, Japan

## Abstract

Schizophrenia and similar psychoses induced by NMDA-type glutamate receptor antagonists, such as phencyclidine (PCP) and ketamine, usually develop after adolescence. Moreover, adult-type behavioral disturbance following NMDA receptor antagonist application in rodents is observed after a critical period at around 3 postnatal weeks. These observations suggest that the schizophrenic symptoms caused by and psychotomimetic effects of NMDA antagonists require the maturation of certain brain neuron circuits and molecular networks, which differentially respond to NMDA receptor antagonists across adolescence and the critical period. From this viewpoint, we have identified a novel developmentally regulated phencyclidine-responsive transcript from the rat thalamus, designated as *prt6*, as a candidate molecule involved in the above schizophrenia-related systems using a DNA microarray technique. The transcript is a non-coding RNA that includes sequences of at least two microRNAs, miR132 and miR212, and is expressed strongly in the brain and testis, with trace or non-detectable levels in the spleen, heart, liver, kidney, lung and skeletal muscle, as revealed by Northern blot analysis. The systemic administration of PCP (7.5 mg/kg, subcutaneously (s.c.)) significantly elevated the expression of *prt6* mRNA in the thalamus at postnatal days (PD) 32 and 50, but not at PD 8, 13, 20, or 24 as compared to saline-treated controls. At PD 50, another NMDA receptor antagonist, dizocilpine (0.5 mg/kg, s.c.), and a schizophrenomimetic dopamine agonist, methamphetamine (4.8 mg/kg, s.c.), mimicked a significant increase in the levels of thalamic *prt6* mRNAs, while a D2 dopmamine receptor antagonist, haloperidol, partly inhibited the increasing influence of PCP on thalamic *prt6* expression without its own effects. These data indicate that prt6 may be involved in the pathophysiology of the onset of drug-induced schizophrenia-like symptoms and schizophrenia through the possible dysregulation of target genes of the long non-coding RNA or microRNAs in the transcript.

## Introduction

Schizophrenia is a severe psychiatric disorder that occurs in the early stages of life with a high morbidity rate. This common disorder produces various mental disturbances and often results in serious disability due to antipsychotic-resistant symptoms. Consequently, the developments of new treatment and preventive and early diagnostic methods are urgent issues. However, the biological mechanisms of the onset of schizophrenia are still unclear. To obtain an insight into the molecular basis of the onset, we have focused our attention on the fact that both schizophrenia and similar psychoses induced by NMDA-type glutamate receptor antagonists, such as phencyclidine (PCP) and ketamine, or by dopamine agonists, such as amphetamines and cocaine, usually emerge after adolescence. Moreover, adult-type behavioral disturbances following the application of NMDA receptor antagonists or dopamine agonists in rodents, which are recognized as models of schizophrenia, are observed after a critical period at around 3 postnatal weeks [1]–[3]. These clinical and experimental observations suggest that schizophrenic symptoms and psychotomimetic effects of NMDA receptor antagonists and dopamine agonists may require the maturation of certain brain neuron circuits and molecular networks specifically dysregulated in schizophrenia and their animal homologues. The molecules composing the above systems should differentially respond to NMDA receptor antagonists or dopamine agonists across adolescence or the critical period.

We therefore have been exploring hypothetical genes that are developmentally regulated and PCP- or methamphetamine-responsive as candidate schizophrenia-related molecules in the rat or mouse brain. These screening analyses have been performed in the neocortex and thalamus because: (i) neurochemical, neuropathological, neurophysiological, and brain imaging studies *in vivo* or in the postmortem brains of schizophrenic patients have consistently highlighted the malfunctions of neural circuits within and/or between the prefrontal and temporal cortex, and other neocortical regions, and thalamic nuclei [4]–[10]; and (ii) the neocortex, thalamus, and their connections have been shown to be major targets for the therapeutic actions of antipsychotic drugs or psychotomimetic effects of NMDA receptor antagonists or dopamine agonists in humans [11]–[14] and experimental animals [15]–[18]. We have identified such candidate genes including CCN1 [19], SAP97 [20], and Lmod2 [1], and a novel gene mrt1 encoding sorting nexin proteins with PX-, PDZ-, and SH-domains [21] from the rat neocortex or thalamus by employing a differential cloning technique or DNA microarray. Through studies on these genes by means of molecular genetics and mouse gene manipulation, we have demonstrated a significant associaiton of SAP97 gene and schizophrenia [22], [23].

In the present study, to advance our knowledge of the molecular cascades involved in the pathophysiology of the onset of schizophrenia, we searched in the thalamus for a novel type of transcript that exhibits expressional changes following the systemic administration of PCP after (PD50), but not before (PD8), the critical period of the psychotomimetic effects of PCP by employing DNA microarray and the RT-PCR technique. We report here a long non-coding RNA that meets these criteria and includes at least 2 micro-RNA sequences.

## Materials and Methods

### Animals

The Committee for Animal Experiment Ethics of Tokyo Medical and Dental University approved the present animal experiments that we performed in strict accordance with the guidance of the University. In this study, we used male Wistar rats (ST strain, Clea Japan, Tokyo, Japan) at postnatal day (PD) 8 (15–25 g), 13 (20–30 g), 20 (35–45 g), 26 (60–80 g), 32 (100–120 g), and 50 (200–260 g). The animals were housed at 24.0±0.5°C under a 12 hour light-dark cycle. Food and water were provided ad libitum.

### Chemicals

Drugs used in this study were obtained from the following resources and administered to animals as described before [1]. Astellas Pharma Inc. (Tokyo, Japan) generously synthesized and donated PCP hydrochloride. We purchased methamphetamine (MAP) hydrochloride from Dainippon Sumitomo Pharma Co., Ltd. (Osaka, Japan). PCP and MAP have been stored and used for our experiments under official permission by the Tokyo Metropolitan Bureau of Public Health. The other chemicals used were of ultrapure quality and commercially available. We dissolved PCP hydrochloride, MAP hydrochloride, and MK-801 hydrogen maleate (dizocilpine hydrogen maleate; [5R, 10S]-[+]-5-methyl-10, 11-dihydro-5Hdibenzo [a,d] cyclohepten-5,10-imine; Sigma-Aldrich) in saline for subcutaneous (s.c.) injection. Haloperidol (HAL) was dissolved in 0.15% tartaric acid and titrated with 0.05 M NaOH to approximately pH 5.0 as previously described [1]. We pretreated some animals with HAL injected intraperitoneally (i.p.) 30 min before PCP [1]. Control animals were administered with the same volume of saline or vehicle. The doses of the above drugs always refer to the free bases, and were determined so as to elicit typical behavioral and biochemical effects [1], [24]–[26].

### Tissue and Total RNA Preparation

Rats were sacrificed by cervical dislocation 1, 1.5, 2, 3, 6, or 24 hours after the injection of various drugs or saline. According to our routine methods [1], we rapidly dissected out the thalamus, neocortex (the dorsal part of the cerebral cortex divided along the rhinal fissure), other discrete brain regions, and peripheral organs on ice, frozen in liquid nitrogen, and stored at −80°C until use. Total RNA was extracted from these frozen tissues by using an RNeasy Midi Kit (Qiagen, GmbH, Hilden, Germany). We verified the quality of RNA using gel electrophoresis (Bioanalyzer, Agilent Tecchnologies, Santa Clara, CA, USA).

### DNA Microarray

To identify the development-dependent PCP-responsive genes in the thalamus, DNA microarray analysis was performed using the Affymetrix Rat Genome 230 2.0 Array (Affymetrix, Santa Clara, CA, USA) by applying the experimental procedures previously described in our paper by Takebayashi et al. [1]. The array system included 31,000 probe sets capable of analyzing the expression level of over 30,000 transcripts and variants from over 28,000 well-substantiated rat genes. Further details can be referred at http://www.affymetrix.com [1].

**Figure 1:**
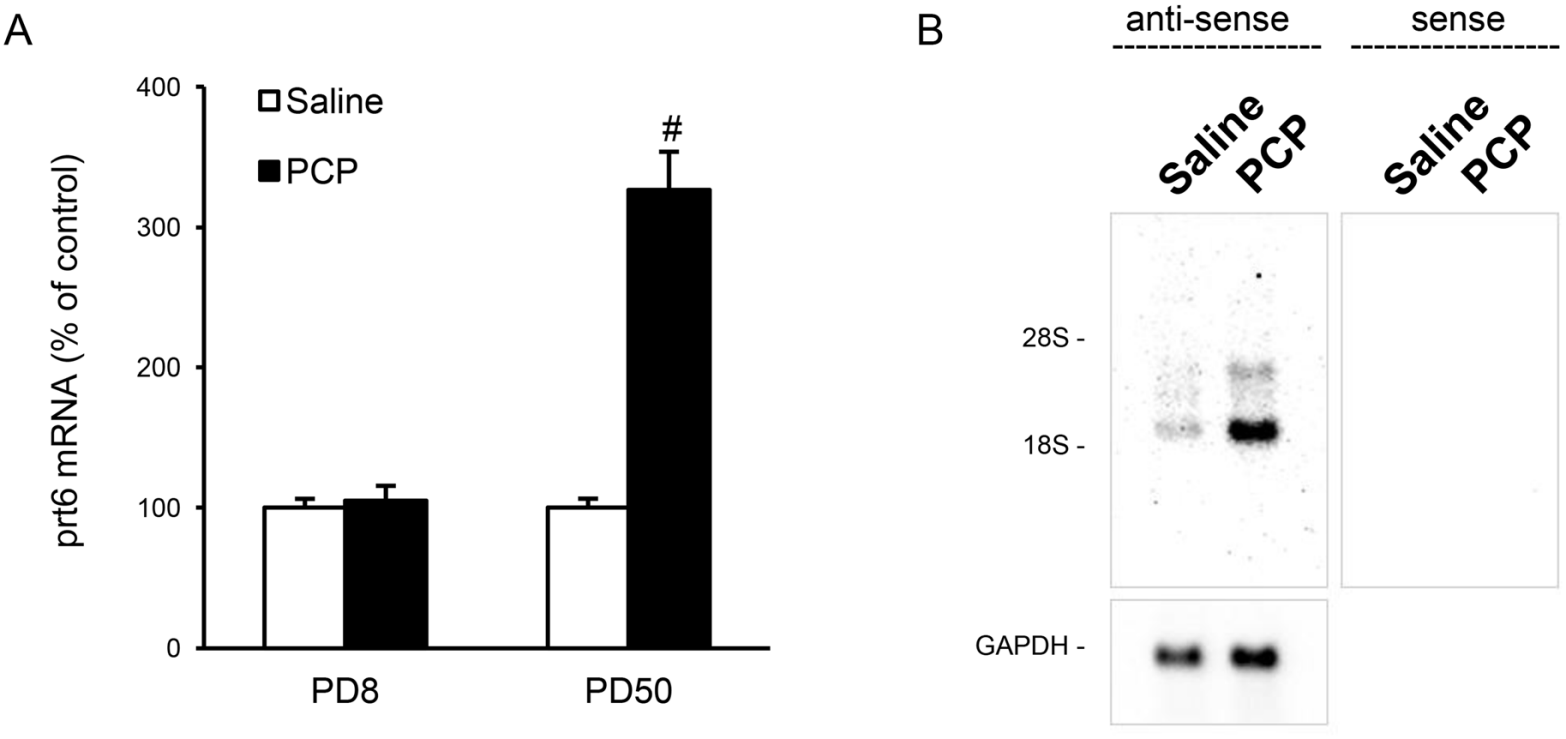
Differential effects of acute PCP injection on *prt6* RNA expression in the infant and adult rat thalamus. (**A**) Expression levels of *prt6* RNA 60 min after PCP administration were determined. Results are the means with S.E.M. of data (the individual ratios of expressional levels of *prt6* to those of GAPDH (*prt6*/GAPDH)) obtained from 8 rats per group: Infant rats (PD8), saline 100±6% (prt6/GAPDH: 0.455±0.029), PCP 105±11%; young adult rats (PD50), saline 100±6% (*prt6*/GAPDH: 0.734±0.047), PCP 327±27%^#^, ^#^p<0.01 vs saline-treated controls; p>0.05 vs saline-treated controls. ^#^P<0.01 vs. respective saline-treated controls. NS, no significant difference. (**B**) Northern blot analysis of *prt6* RNA in the poly(A)^+^ RNA fraction (2 µg) prepared from the thalamus of adult rats. Hybridization of a selective probe to *prt6* transcripts against the sequence near their 3′-side (Figure 2A) indicated two major bands at 1.9- and 3.0-kb in the lanes of the thalamic tissues from saline- and PCP-treated adult rats. These signals were up-regulated by acute PCP administration.

For this selection process by DNA array, we prepared the below four experimental groups of rats, as previously reported [1]: five saline-injected control rats at PD50; five PCP (7.5 mg/kg, s.c.)-treated rats at PD50; five saline-injected control rats at PD8; five PCP (7.5 mg/kg, s.c.)-treated rats at PD8. In each experimental group, we pooled equal amounts of total RNA individually isolated from the five animals per experimental group as mentioned before [1]. We conducted cDNA synthesis, cRNA labeling, hybridization and scanning according to the manufacturer’s instructions (Affymetrix). The candidate genes were first screened by a single microarray comparison of the above four sets of pooled cDNAs from the four respective experimental groups. The data that the 3′/5′ ratios of glyceraldehyde-3-phosphate dehydrogenase (GAPDH), hexokinase, and β-actin of all four samples used were less than 1.2, 2.2, and 3.0, respectively, met the sample quality standard confirmed by a signal value ratio of less than 3. Excellent inter- and intra-platform reproducibilities of the Affymetrix microarray (more than 88% and 90%, respectively) were shown elsewhere [27], supporting the reliability of the assay system. We further verify the results obtained from the microarray assay by conducting quantitative RT-PCR analyses and/or Northern blotting of the candidate transcripts screened from the first single microarray in individual (Figure 1A) or pooled (Figure 1B) samples.

### Analysis of the Full-length Structure of EST

To determine the full-length structure of the candidate EST (expression sequence tag) designated as PCP responsive transcript 6 (*prt6*) that is differentially regulated by PCP between PD 8 and 50, we performed 5′ rapid amplification of cDNA ends (RACE) PCR with one microgram of total RNA extracted from the thalamus the using the SMART RACE cDNA amplification kit according to the manufacturer’s protocol (TAKARA Bio/Clontech, Kusatsu, Japan). The following primers were used for the 5′ RACE-PCR: Fragment A, 5′-GGATATAACAAAAGCATGCCCACCAC-3′; Fragment B, 5′-GGCGGCCCGGGCAGTAAGCAGTCTA-3′.

### Quantitative RT-PCR

The present quantitative RT-PCR analyses were performed according to the methods described previously [1]. The total RNAs extracted from the brain tissues were further treated with DNase using the RNase-Free DNase Set (Qiagen). Total RNA (1–2 µg) was reverse transcribed with Oligo(dT)_20_ using Superscript III (Invitrogen, Gaithersburg, MD, USA). We accomplished real-time quantitative RT-PCR on a LightCycler (Roche, Mannheim, Germany) using SYBR Premix Ex Taq (TAKARA Bio) according to the following manufacturer’s protocol. PCR primers were designed with Primer Express software (Applied Biosystems, Foster City, CA, U.S.A.) with default parameters. We normalized the expressional levels of each gene to that of GAPDH. Relative mRNA abundance was calculated using the standard curve method as described in the Applied Biosystems manual. In some experiments to evaluate the regional distribution of the candidate transcript, *prt6*, in the brain, semi-quantitative RT-PCR was conducted on a LightCycler. Reaction products were electrophoretically separated on 10% acrylamide gel, and visualized by staining with SYBR Green I. The primer sequences used are as follows: *prt6*: 5′-GCATGCTTAAGGTCCTGGCA-3′ (2575–2594) and 5′-CCCCACCACAGCTTAATTCGT-3′ (2605–2625); GAPDH (Accession No. NM_017008) 5′-ACATCATCCCTGCATCCACT-3′ (1457–1476) and 5′-GGGAGTTGCTGTTGAACTCA-3′ (1695–1714). Primers used for real-time RT-PCR of *prt6*: position of −508 to −458: 5′-TGGTAGTGATAGCATCGCGG-3′ and 5′-ACCAGACAGCCCTATGACGC-3′; position of 39 to 98: 5′-TAAGGACCCACGGTCAGTCG-3′ and 5′-CTCTTTGCCCTCCCATGGT-3′; position of 235 to 285: 5′-CCCAAATGCAGACGCAGAC-3′ and 5′- GCTCGGTTCTCCGCTTGAT-3′, position of 684 to 739: 5′-CCACTGGGACATCTTTGACGT-3′ and 5′-GGACAGACACGCGACCGT-3′; position of 1792 to 1842: 5′-CACAGAGGCTGCAGGTAAACG-3′ and 5′-CAGATGCTCTCAACAGCCCCA-3′.

### Northern Hybridization

Northern hybridization of prt6 transcripts was carried out as follows according to the methods described before [1]. We purified two micrograms of Poly(A)^+^ RNA from the rat thalamus using an oligo(dT)-cellulose column (Amersham, Buckinghamsire, UK). The Poly(A)^+^ RNA extract was separated by formaldehyde/1.0% agarose gel electrophoresis and transferred to a Hybond-XL membrane (Amersham). A 298-bp DNA fragment corresponding to the nucleotide positions 2463–2760 of the rat *prt6* cDNA was subcloned into pGEM-T easy Vector. Plasmids were linearized, and digoxigenin (DIG)-labeled RNA probes were prepared by in vitro transcription using T7 and SP6 RNA polymerase. Hybridization was performed at 68°C in DIG-Easy Hyb buffer (Roche). The membranes were washed in 2× SSC/0.1% SDS at room temperature, and 0.1× SSC/0.1% SDS at 68°C. Finally, we immunodetected the hybridized probes with anti-DIG-AP, and then visualized with a chemiluminescence substrate (CDP-Star, Roche). We used pre-made Rat Multiple Tissue Northern Blot (TAKARA Bio) in some experiments.

### Statistical Analysis

Statistical analysis of the present data was achieved as presented previously [1]. Results are given as the mean with S.E.M. of the data. For comparison between the two groups with homogeneous variants, statistical evaluations were performed with the unpaired two-tailed Student’s *t*-test. Significant differences among more than three groups were estimated by the Kruskal-Wallis test followed by Schffé post-hoc test.

### Nucleotide Sequences

The DDBJ/GenBank/EMBL accession numbers for the primary nucleotide sequence of rat *prt6* Fragment A and fragment B are AB899790 and AB899791, respectively.

## Results

### Identification of *Prt6* as a Developmentally Regulated PCP-responsive Transcript by a DNA Microarray Method, RT-PCR Assay, and Northern Blot Analysis

The DNA array screening revealed that candidates for the transcripts differentially regulated by PCP in the thalamus between infant and adult rats include the EST of Affymetrix ID: 1392108_at, which is designated as PCP-responsive transcript 6 (*prt6*). We selected the EST for subsequent analyses because: (i) it was one of the two transcripts that showed a more than two-fold increase in expression levels after PCP treatment compared to saline treatment, and (ii) the other transcript has already been investigated [1] The development-dependent upregulation of the candidate transcripts including the EST sequences was confirmed by RT-PCR and Northern blot analysis using the primer set and RNA probe, respectively, designed in the EST sequences. The quantitative RT-PCR assays in individual, but not pooled, samples indicated that PCP induced no or a marked increase in *prt6* expression in the thalamus at PD 8 and 50, respectively (**Figure 1A**). On Northern blot analysis of 2 µg of poly(A)^+^ RNA from the adult rat thalamus, two distinct bands at 1.9 and 3.0 kb were detected with an RNA probe in an anti-sense-specific fashion (**Figure 1B**). The signal intensity of both bands was augmented after the subcutaneous PCP injections. The 1.9-kb band exhibited a much more intensive signal than the 3.0-kb band.

### Structural Analysis of *Prt6* RNA Products

**Figure 2:**
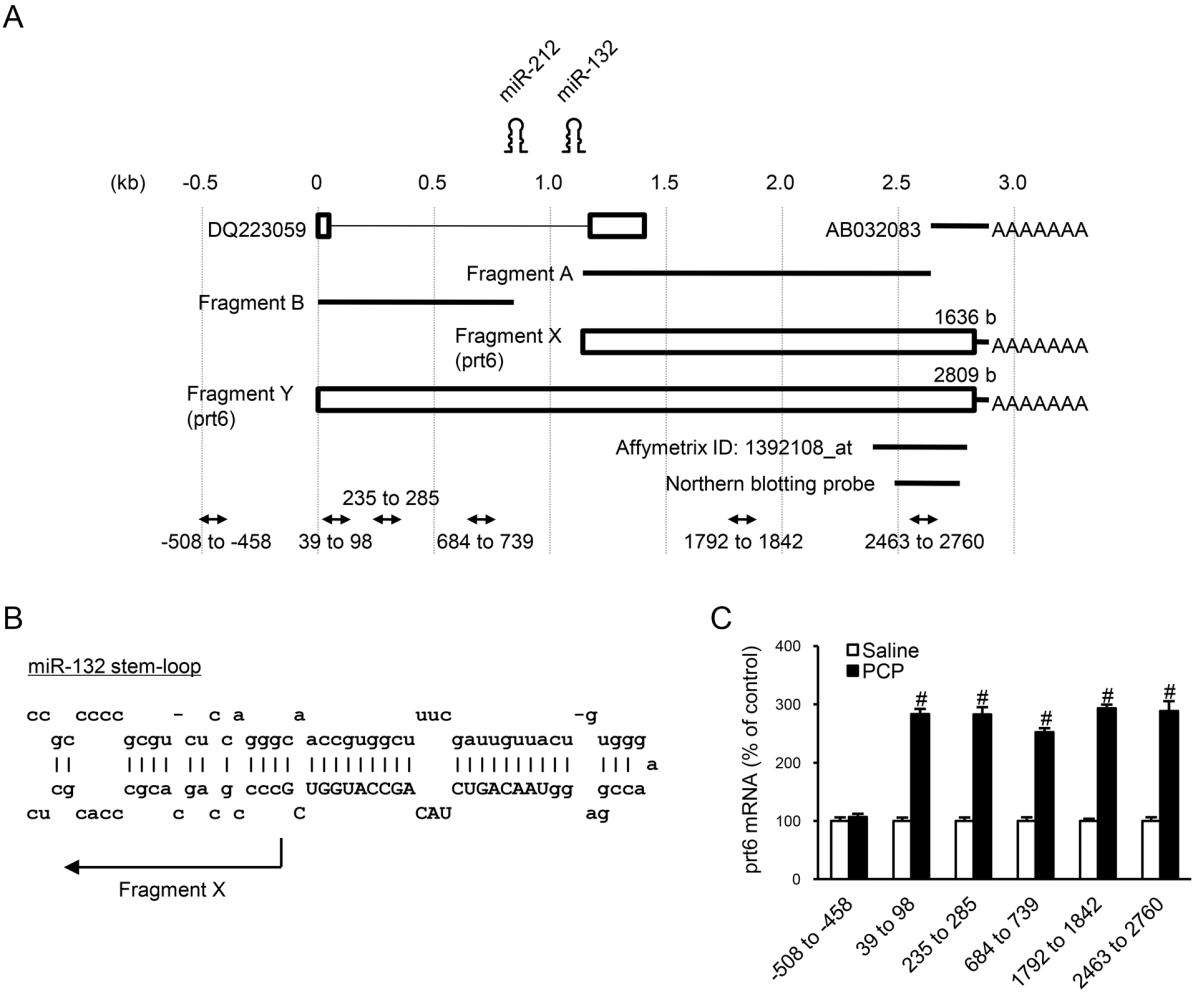
Cloning and expression of rat *prt6*. (**A**) Structures of *prt6* cDNAs. The schematic presentation of *prt6* transcripts is illustrated based on the data obtained by RACE-PCR and database analyses. The two *prt6* transcripts of 1636 bases (Fragment X) and 2809 bases (Fragment Y), shown as open boxes, correspond to the lower and upper bands detected by Northern blot, respectively. Bold lines of Fragments A and B indicate positions of the cDNA fragments detected by 5′-RACE-PCR. (**B**) The 5′ end of Fragment X is located exactly adjacent to the 3′ end of preRNA for miR-132. Capital letters indicate the nucleotide sequences of mature miR-132. (**C**) Expression levels of the various parts of the putative *prt6* RNA 60 min after acute PCP administration were determined by RT-PCR with the primer sets for amplification of the respective parts indicated in Figure 2A. Results are the means with S.E.M. of data (the individual ratios of expressional levels of *prt6* to those of GAPDH (*prt6*/GAPDH)) obtained from 8 rats per group: −508 to −458, saline 100±6% (prt6/GAPDH: 0.859±0.052), PCP 107±6%, p>0.05 vs saline-treated controls; 39 to 98, saline 100±6% (*prt6*/GAPDH: 0.591±0.010), PCP 283±9%^#^, ^#^p<0.01 vs saline-treated controls; 235 to 285, saline 100±6% (*prt6*/GAPDH: 0.576±0.034), PCP 283±13%^#^, ^#^p<0.01 vs saline-treated controls; 684 to 739, saline 100±6% (*prt6*/GAPDH: 0.634±0.040), PCP 252±7%^#^, ^#^p<0.01 vs saline-treated controls; 1792 to 1842, saline 100±4% (*prt6*/GAPDH: 0.515±0.020), PCP 293±6%^#^, ^#^p<0.01 vs saline-treated controls; 2436 to 2760, saline 100±6% (*prt6*/GAPDH: 0.527±0.033), PCP 289±16%^#^, ^#^p<0.01 vs saline-treated controls. ^#^P<0.01 vs. respective saline-treated controls.

We next examined the detailed primary structure of the *prt6* RNAs. Because our initial database search revealed that the sequence of the candidate EST designated as *prt6* overlaps with that of AB032083 containing the typical polyadenylation signal (AATAAA) (**Figure 2A**), we performed 5′ RACE-PCR [28]. We first identified and confirmed a primary structure of *prt6*-Fragment A (**Figure 2A**). Based on the further database search, the presumable *prt6*-Fragment X with 1636 bases was speculated to be a non-coding transcript without significant ORF (**Figure 2A**). With consideration of poly(A) addition, the size of this transcript coincided with that of the lower band predominantly detected in the Northern blot analysis (**Figure 1B**). However, based on the rat genomic information, the upstream regions of the 5′ end of this transcript lack the known core promoter elements, such as the TATA box [29]. Instead, we speculated presence of a tandem array of microRNAs (miRNAs), miR-212 and miR-132, located upstream of the 5′ end of Fragment X (**Figure 2A**). Therefore, we next performed 5′ RACE-PCR using a second specific primer located upstream of the 5′ end of miR-212. We consequently isolated and determined the nucleotide sequence of *prt6*-Fragment B. Based on these observation, we speculate the existence of *prt6*-Fragment Y transcript with 2809 bases without any significant ORF (**Figure 2A**). The size of this transcript with additional poly(A) tail, coincided with that of the upper band detected in Northern blot analysis (**Figure 1B**). The transcriptional initiation site of this transcript is almost the same as that of a non-coding miR132-associated transcript, DQ223059, previously reported in the rat [30], and the canonical TATA box (TATAAA) is located at 25 bp upstream of the start site (**Figure 2A**).

*In silico* overall structural analysis of *prt6* suggests that Fragment Y corresponds to a presumable long primary transcript as pri-RNA of miR-132 and -212 and of their 3′ flanking fragment. The 5′ end of Fragment X (and thereby Fragment A) is located exactly at the flanking region of 3′ end of preRNA of miR-132 (**Figure 2B**). Based on the below observations, these structural features lead to the suggestion that miR-132 and -212 could be derived from these *prt6* RNA products. Thus, recent studies have revealed that the primary transcripts of miRNA genes, pri-miRNAs, are usually several kilo-bases in length and contain single or multiple local hairpin structures (see Kim, 2005 for a review [31]). Subsequently, the stem-loop structure is processed by the nuclear RNase III Drosha to yield the precursor of miRNA [31]. Moreover, the remnants, termed flanking fragments, which have been considered to be degraded in the nucleus, have been cloned as EST [31].

The quantitative real-time RT-PCR assay was performed at 5 different positions covering the pre dicted primary transcript, *prt6*-Fragment Y. We observed a similarly augmented expression of the transcripts in all regions examined after acute PCP injection (7.5 mg/kg. s.c.) (**Figure 2C**). On the other hand, the PCP treatment failed to affect the expressional level with the primer sets designed at upstream of the hypothesized 5′ end of Fragment Y. These data support the presence of the RNAs originated from the presumed primary transcript, *prt6*-Fragment Y, and its induction by PCP at the transcriptional initiation.

### Basal Expression of *Prt6* in Various Organs and Brain Regions

**Figure 3:**
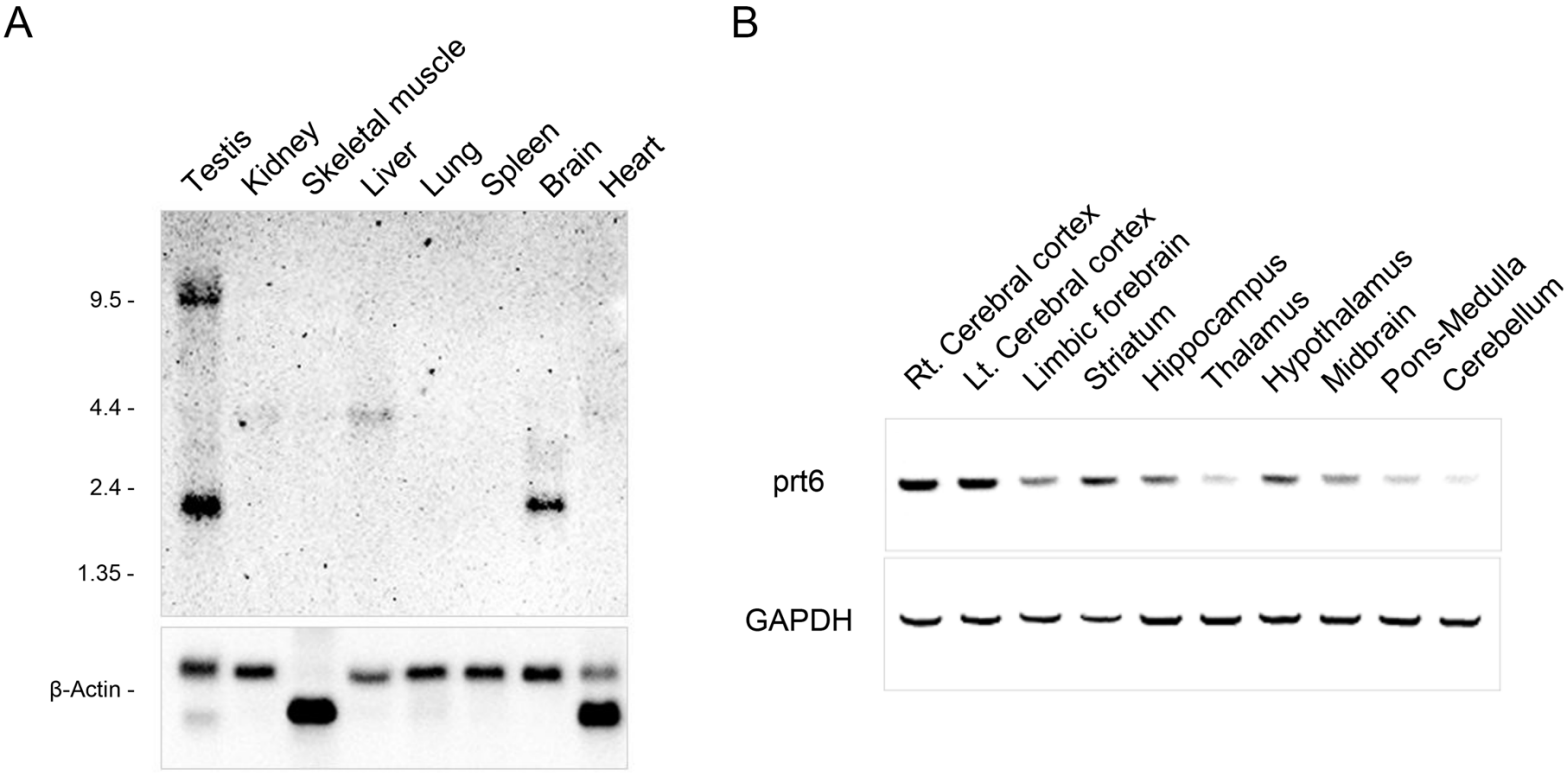
The basal expression of *prt6* in the rat brain and peripheral tissues. (**A**) Northern blot analysis of *prt6* RNA in the poly(A)^+^ RNA fraction (2 µg) prepared from various adult rat tissues. The *prt6* transcripts were identified in the brain and testis. The testis contained transcripts of 9.5 and 1.9 kb. (**B**) Regional distribution of *prt6* RNA in the brain as basal expression determined by RT-PCR.

Northern blot analyses indicated that the 1.9- and 3.0-kb RNA products of *prt6* were predominantly expressed in the brain and testis, while another larger transcript is present, at least, in the liver (**Figure 3A**). The testis also sowed transcripts of 9.5 kb. We further examined the regional distribution of *prt6* in the brain of young adult rats by RT-PCR. The expression of *prt6* RNA products was marked in the forebrain areas, such as the cerebral cortex, limbic forebrain containing the septum, olfactory tubercle and nucleus accumbens, striatum, and hippocampus, followed by the hypothalamus and midbrain at a moderate level, and low in the thalamus, pons-medulla, and cerebellum (**Figure 3B**).

### Effects of Acute PCP Administration on *Prt6* Expression in the Thalamus of the Developing Rats

**Figure 4:**
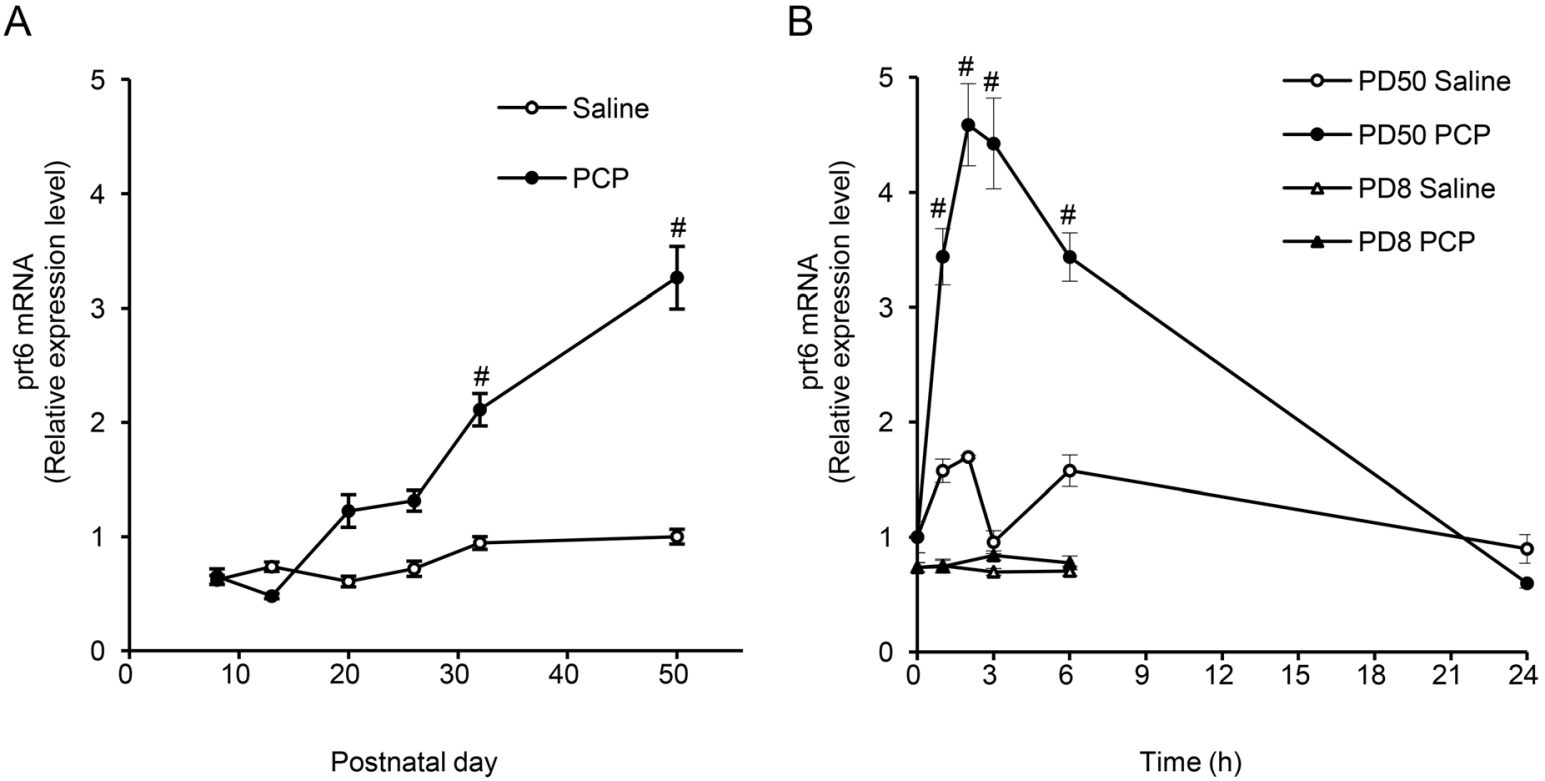
Effects of acute PCP injection on *prt6* expression in thalamus of developing rats and their time-course. (**A**) Effects of acute subcutaneous administration of PCP at 7.5 mg/kg on *prt6* gene expression in the rats at PD8, PD13, PD20, PD26, PD32, and PD50. Expression level*s* of *prt6* mRNA were determined by RT-PCR 60 min after PCP administration. Results are the means with S.E.M. of data (the individual ratios of expressional levels of *prt6* to those of GAPDH (prt6/GAPDH)) obtained from 5 or 6 rats per group: Infant rats (PD8), saline, prt6/GAPDH 0.620±0.040; PCP, 0.650±0.067. ^#^P<0.01 vs. respective saline-treated controls. (**B**) Time-course of *prt6* mRNA expression after acute subcutaneous PCP administration in the adult (PD50) and infant (PD8) rat thalamus. Results are the means with S.E.M. of data obtained from 5 or 6 rats per group (prt6/GAPDH): Infant rats (PD8), zero-hour 0.737±0.040; Adult rats (PD50), zero-hour 1.001±0.040; PCP, 0.650±0.067. The zero-hour group of rats received neither PCP nor saline. ^#^ P<0.01 vs. respective saline-treated controls.

The expression levels of thalamic *prt6* following saline injection (control values) remained unchanged from PD 8 to 50 in rats (**Figure 4**). In contrast, PCP treatment (7.5 mg/kg, s.c.) led to a significant elevation in the ratios of RNA levels of *prt6* to those of GAPDH as compared to the saline-treated controls at PD32 and PD50, but not at PD8, PD13, PD20, and PD24, although there was a increasing tendency in prt6 expression in the PCP-treated rats at PD20 and PD24 (**Figure 4A**). These data further verified the differential responses of *prt6* to PCP between PD8 and PD50 observed in the DNA array analysis. At PD32 and PD50, when *prt6* is significantly upregulated, we previously found that PCP induced an adult type of abnormal behavior [1].

The time-courses of the effects of an acute injection of PCP (7.5 mg/kg, s.c.) on the *prt6* expression levels in the thalamus were distinct between infant and adult rats. The thalamic *prt6* RNA expression in the young adult rats (PD50) was rapidly and significantly increased from 1 to 6 hours, peaked at 2–3 hours and returned to the saline-treated levels within 24 h after the acute PCP administration **(** **Figure 4B** **).** However, there were no changes in the *prt6* expression in the thalamus of the infant rats (PD8) up to 6 hours post-injection.

### Effects of Acute PCP Administration on *Prt6* Expression in the Neocortex, Hippocampus, and Thalamus of Young Adult Rats

**Figure 5:**
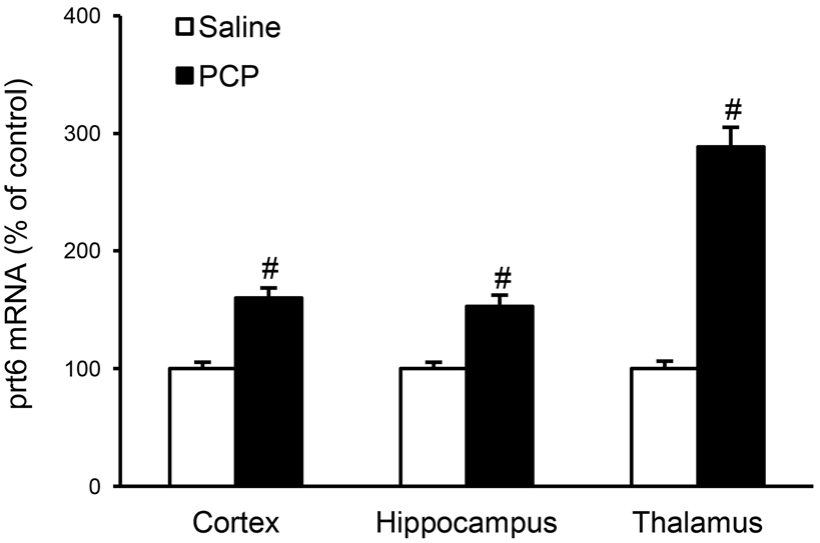
Efects of acute injection of PCP on *prt6* expression in the neocortex, hippocampus, and thalamus of adult rats. Relative expression levels of *prt6* mRNAs (*prt6* to GAPDH mRNA ratio) were determined by the real-time RT-PCR method in the neocortex, hippocampus, and thalamus of the adult (PD 50) rat 60 min after PCP (7.5 mg/kg, s.c.) or saline administration. Results are the means with S.E.M. of data obtained from 8 rats per group and are expressed as a percentage of the values of the respective saline-treated controls: cortex, saline 100±5% (*prt6*/GAPDH: 0.744±0.040), PCP 160±9^#^, ^#^p<0.01 vs saline-treated controls; hippocampus, saline 100±5%, (*prt6*/GAPDH: 0.635±0.045) PCP 153±10^#^, ^#^p<0.01 vs saline-treated controls; thalamus, saline 100±6% (prt6/GAPDH: 0.527±0.034), PCP 289±17^#^, ^#^p<0.01 vs saline-treated controls.

The acute systemic administration of PCP (7.5 mg/kg, s.c.) led to a significant increase in *prt6* expression levels not only in the thalamus but also in the neocortex and hippocampus (**Figure 5**). The magnitude of the increasing effects of PCP was greater in the thalamus than in other brain regions.

### Effects of Acute Administration of NMDA Receptor Antagonists, Dopamine Agonist and Antagonist on *Prt6* Expression in the Thalamus of Adult Rats

**Figure 6:**
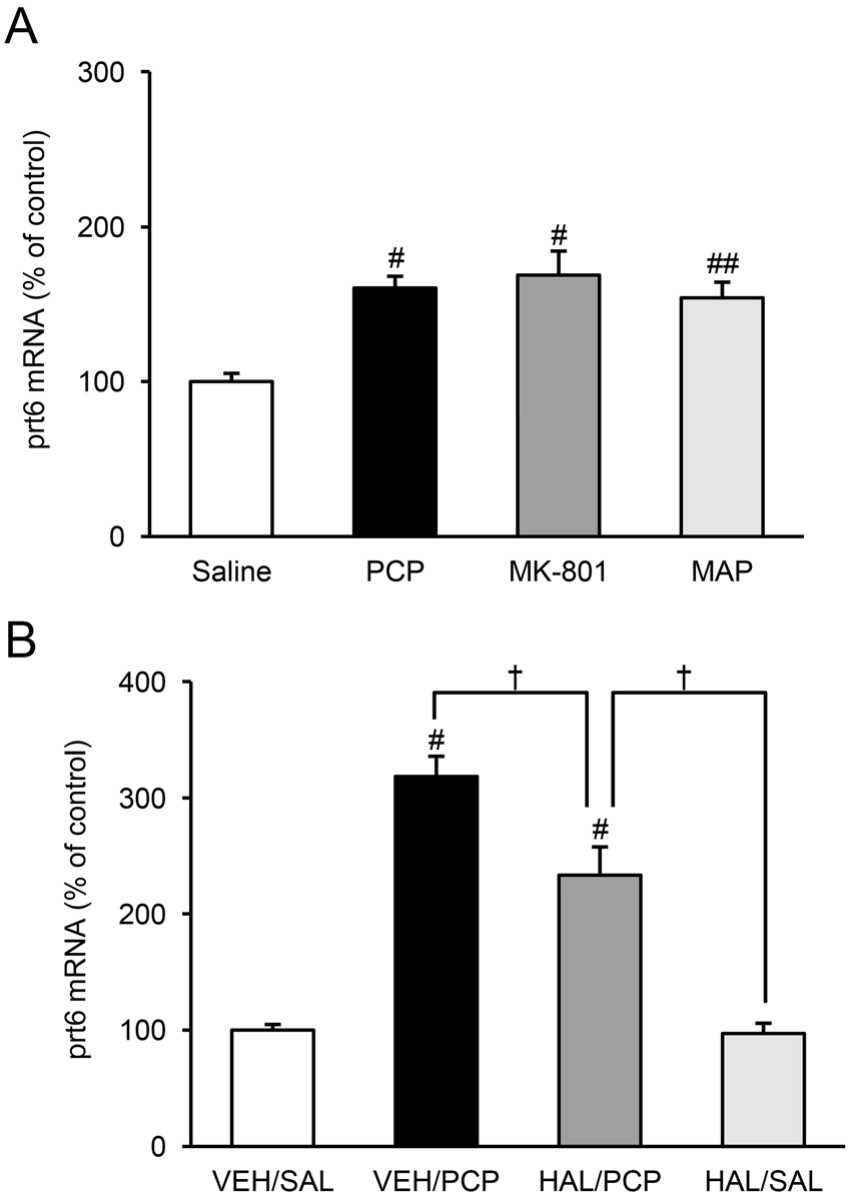
Effects of acute administration of psychotomimetic and antipsychotic drugs on *prt6* mRNA expression in the thalamus. (**A**) Effects of PCP, MK-801 and methamphetamine (MAP) on thalamic *prt6* mRNA. Relative expression levels of *prt6* mRNA in the thalamus of the adult (PD 50) rat (*prt6* to GAPDH mRNA ratio) were assayed by the real-time RT-PCR method 60 min after acute PCP (7.5 mg/kg, s.c.), MK-801 (0.5 mg/kg, s.c.), and MAP (4.8 mg/kg, s.c.) administration. Results are means with S.E.M. of data (*prt6* to GAPDH mRNA ratio) obtained from 6 rats per group, expressed as a percentage of the values of the saline-treated controls: saline (SAL) 100±5% (*prt6*/GAPDH: 0.821±0.042), PCP 161±7%^#^, MK-801 169±16%^#^, MAP 154±10%^##^. ^#^P<0.01, ^##^P<0.05 vs. saline-treated controls. (**B**) Effects of pretreatment with haloperidol (HAL) on PCP-induced upregulation of the thalamic *prt6* mRNA. The adult (PD 50) rats were pretreated with HAL (1.0 mg/kg, i.p.) or vehicle (VEH) 30 min before PCP or saline (SAL) administration, and the relative expression levels of *prt6* mRNA in the thalamus (*prt6* to GAPDH mRNA ratio) were assayed by the real-time RT-PCR method 60 min after acute PCP (7.5 mg/kg, s.c.) or saline injection. Results are the means with S.E.M. of data (*prt6* to GAPDH mRNA ratio) obtained from 6 rats per group and are expressed as a percentage of the values of the vehicle-pretreated and saline-injected controls: vehicle-pretreated saline-injected controls (VEH/SAL) 100±5% (*prt6*/GAPDH: 0.527±0.024), vehicle-pretreated PCP-injected animals (VEH/PCP) 319±17%^#^, haloperidol-pretreated PCP-injected animals (HAL/PCP) 234±24%^#^, haloperidol-pretreated saline-injected animals (HAL/SAL) 97±9%. ^#^P<0.01 vs. VEH/SAL controls. ^†^P<0.01 between VEH/PCP and HAL/PCP animals and between HAL/PCP and HAL/SAL animals. Vehicle (VEH): 0.15% tartaric acid.

To evaluate the pharmacological nature of the PCP-induced upregulation of *prt6*, we studied the effects of another psychotomimetic NMDA receptor antagonist, dizocilpine (MK-801: 0.5 mg/kg, s.c.), a schizophrenomimetic indirect dopamine agonist, methamphetamine (4.8 mg/kg, s.c.), and a typical antipsychotic with a potent D2 receptor blocking action, haloperidol (1.0 mg/kg, i.p.), on *prt6* expression in the rat thalamus. MK-801 and MAP mimicked the effect of PCP of increasing the thalamic *prt6* expression (**Figure 6A**). Pretreatment with haloperidol 30 min before PCP injection partly antagonized the ability of PCP to augment *prt6* expression in the thalamus (**Figure 6B**). These results are consistent with the view that PCP-induced elevation of *prt6* expression might be related to the influence of PCP on dopaminergic and extra-dopaminergic systems following blockade of the NMDA receptor [32], [33].

The magnitude of the PCP-induced increase in *prt6* expression in this experiment, shown in **Figure 6A**, was smaller than in the other experiments in the present study. These differences appear to be due to the higher values of *prt6* expression in the saline-treated animals in this observation compared to the other sets of saline-injected animals.

## Discussion

In the present study we disclosed that the gene designated prt6, encoding non-coding RNAs with the sequences of EST 1392108_at (Affymetrix), miR-212, and miR-132, is development- and PCP-regulated gene in the rat thalamus, and that the schizophrenomimetic NMDA receptor antagonist elicits a significant increase in the expression levels of *prt6* at PD32 and 50, but not PD8, 13, 20 and 25. *prt6* RNAs are strongly expressed in the brain and testis, and are also upregulated by PCP in the neocortex and hippocampus of young adult rats at PD50. The PCP-induced upregulation of thalamic *prt6* RNAs in the adult period is mimicked by other schizophrenomimetics, dizocipine and MAP, and partly attenuated by the D2-selective dopamine receptor antagonist haloperidol. These structural, developmental, and pharmacological features of *prt6* suggest that the non-coding RNA products of *prt6* or their target genes could be dysregulated in the late-developing abnormal behavior induced by PCP.

There has so far been no report demonstrating that PCP or other NMDA receptor antagonists alter the expression of long non-coding RNA in mammalian brain tissues. The PCP induction of thalamic *prt6* at PD50 does not appear to be a non-specific phenomenon, because we previously failed to find any changes in the levels of Tmod1 and Tmod2 mRNAs in the thalamus after the same PCP regimen [1]. The lack of *prt6* upregulation before the critical period for the adult-type PCP-induced abnormal behavior is simply caused by the possible developmental changes in the pharmacodynamics of PCP or other psychotomimetic drugs. However, this is likely to be ruled out based on the following observations: (i) like those of another thalamic developmentally regulated PCP responsive gene, Lmod2 [1], thalamic *prt6* expression levels were unaffected by PCP even up to 6 hours post-application in the infant rat (PD8) while the prominent increase in those of the adult rat was observed during the same period (**Figure 4B**); (ii) a similar time-course of the acute PCP-induced increase in c-*fos* gene expression was seen in various brain areas of the rat at PD 8 and 50 [34]; and (iii) MAP injection produced a marked elevation in *c-fos* gene expression in the striatum of the infant and adult rats [35]. The immediate early gene-like response of *prt6* to PCP in the young adult period (**Figure 4B**) might be triggered by the cyclic-AMP responsive elements (CREs) located in the 5′-side of respective microRNA regions of the *prt6* transcript (**Figure 2A**).

The developmental changes in the PCP-induced *prt6* expression in the thalamus are more likely to reflect the functioning of certain neuron circuits influenced by the thalamic and/or extra-thalamic NMDA receptor activity. The development-dependent and PCP-induced upregulation of *prt6* appears to be linked to the psychotomimetic effects of PCP due to reduced NMDA receptor-mediated glutamate transmission and augmented dopamine transmission. Thus, significant upregulation occurs only after a critical period for the adult type of PCP-induced abnormal behavior in the rat (**Figure 4A**; [1]). Moreover, the selective NMDA antagonist MK-801 and the indirect dopamine agonist MAP enhanced thalamic *prt6* expression. Finally, the effect of PCP to increase *prt6* non-coding RNA levels was partly, but significantly, attenuated by a selective D2 dopamine receptor antagonist, haloperidol (**Figure 6**). From these antipsychotic-responsive and -resistant profiles of the PCP-related induction of *prt6* dysregulation, we can hypothesize that the non-coding RNA products of *prt6* function as regulatory factors in molecular cascades that are impaired in dopamine-associated positive symptoms, and NMDA receptor-related dopamine-uncoupled negative symptoms, and cognitive deficits in PCP psychosis and schizophrenia [36]–[38].

There was a prominent regional difference among the basal expressional levels of *prt6* transcripts in the rat brain that were relatively low in the thalamus but high in the neocortex and hippocampus (**Figure 3B**). By contrast, the thalamus displayed much larger percentages of increase in prt6 mRNA expression after PCP injection than the two brain areas (**Figure 5**), suggesting a significant role of thalamic *prt6* in psychotomimetic effects of PCP. Indeed, based upon the extracellular recordings and brain activity mapping by *c-fos* gene expression, Santana et al. [39] provided convergent evidence indicating that the thalamus may be one of the primary sites of psychotomimetic action of PCP.

It is generally accepted that non-coding RNAs such as micro-RNAs control the expression of target genes by interacting with their anti-sense sequences against the restricted nucleotide sequences of the respective non-coding RNAs. More recently, evidence has accumulated indicating that long non-coding RNAs contribute to structural or chemical modifications of chromatin and gene activation besides the well-known repression of gene expression [40]. The non-coding RNA, *prt6*, could exert regulatory roles in gene expression in its full length and/or its putative non-coding variants, as shown in **Figure 2A**. From Northern blot analysis of the thalamic poly-A RNAs, PCP seems to chiefly augment the expression levels of the 3.0- and 1.9-kb RNAs (**Figure 1B**), Fragment-Y and -X, respectively (**Figure 2**). The former changes are consistent with quantitative RT-PCR studies using the different primer sets for the 5 distinct parts throughout the putative *prt6* non-coding RNA showing a similar magnitude of PCP-induced increase in the expression level of each PCR product (**Figure 2C**). These observations also support the presence of approximately 3.0-kb transcripts of *prt6* (**Figure 2A**).

The upregulated 3.0-kb (Fragment Y) product could act in the brain, at least in part, as a long primary transcript (pri-miRNA) for miR-132 and/or miR212, because the sequences of these micro-RNAs are tandemly located within the transcript. In agreement with this, our recent DNA microarray experiments showed that PCP administered with the same regimen as in the present study suppresses the neocortical expression of the predicted targets of these two miRNAs, *PAIP2A* and *H2AFZ* (unpublished data), suggested by sequence analyses (miRanda and TargetScan) of miR-132 and miR212. Because these micro-RNAs have been shown to play important roles in the development, maturation, and function of neurons [41], [42], the minimal and marked developmental changes in the basal expression of *prt6* (**Figure 4**) and miR-132 [42], respectively, could be accounted for by the possible alterations in the processing of various non-coding RNAs related to *prt6* during embryonic and pre-weaning periods.

The functional link between the NMDA receptor and *prt6* appears to be further supported by the observations of Matsuo et al. (2000) [28] that the RM2 transcript (**Figure 2**), which shares the nucleotide sequence in the 3′-portion of the *prt6* transcript, was upregulated following LTP induction by high-frequency stimulation in the rat hippocampus in an NMDA receptor antagonist-reversible manner. In terms of the NMDA receptor function-related and development-dependent schizophrenomimetic-responsive nature of *prt6*, it should be noted that miR-132 and miR-212, the sequences of which are found in *prt6* RNA (**Figure 2**), have been reported to be dysregulated in the prefrontal cortex of postmortem brains from schizophrenic patients [43]–[45]. This dysregulation could be attributed to alterations in the expression of *prt6* RNA and/or its cleavage mechanisms.

In conclusion, the current data demonstrate that non-coding RNA-type transcripts of the *prt6* gene show developmentally regulated responses to a schizophrenomimetic PCP in an NMDA and D2 receptor-related fashion. The late developing pharmacological feature and presence of the known micro- and long non-coding RNA sequences in the transcripts suggest that the molecular pathways that may comprehensively control the maturation and balance of behavioral and mental functions could include the *prt6* non-coding RNA regulatory system, consisting of a certain hierarchy of non-coding RNAs with a different size and structure. A further investigation to clarify the mechanisms of cleavage or editing of the *prt6* transcripts and their pathological alterations in schizophrenic brains is now in progress.
